# Effects of Expansion Temperature on Fiber Characteristics, Feed Processing Quality, and Growth Performance of Pigs Fed *Cyperus esculentus* Meal

**DOI:** 10.3390/ani16172714

**Published:** 2026-09-01

**Authors:** Jie Zeng, Zheng Peng, Changhong Xia, Xiaokai Lu, Fengchi Liu, Hanbing Zhou, Daiya Wang, Hao Cheng, Si Gao, Zhili Qi

**Affiliations:** Department of Animal Nutrition and Feed Science, College of Animal Science and Technology, Huazhong Agricultural University, Wuhan 430070, China; zengjie1999@webmail.hzau.edu.cn (J.Z.); pengzheng@webmail.hzau.edu.cn (Z.P.); xch001020@163.com (C.X.); lu1399425597@gmail.com (X.L.); 15327408796@163.com (F.L.); zhouhanbing@webmail.hzau.edu.cn (H.Z.); dywang0529@163.com (D.W.); ch3208556806@gmail.com (H.C.); gaosi@mail.hzau.edu.cn (S.G.)

**Keywords:** extrusion, *Cyperus esculentus* meal, physicochemical characteristics of fibre, pellet feed quality, growth performance

## Abstract

Extrusion processing can modify dietary fibre structure and enhance feed quality, but its temperature-dependent effects on *Cyperus esculentus* meal (CEM) remain unclear. This study evaluated the effects of varying extrusion temperatures (110–125 °C) on fibre properties, pellet quality, and growth performance in nursery pigs. A total of 527 pigs were randomly assigned to six dietary treatments for 21 days: a corn–soybean meal control, a diet containing 20% unextruded CEM, and four diets containing 20% extruded CEM (ECEM) processed at 110, 115, 120 or 125 °C. Extrusion at 110–120 °C optimally improved fibre functionality, reducing neutral detergent fibre by approximately 12%, while enhancing water- and oil-holding capacities. Pellet quality was substantially improved, with hardness increasing from 29.5 N to 52.7 N and fines content dropping from 5.4% to 1.9% at optimal temperatures. Growth performance and diarrhoea incidence were unaffected by dietary treatments. Notably, ECEM increased faecal microbial diversity, enriching beneficial bacteria while suppressing potential pathogens. In conclusion, extrusion at 110–120 °C effectively transforms CEM into a functional ingredient that enhances feed quality and gut health without compromising growth, offering a practical strategy to reduce reliance on conventional grains in nursery pig diets.

## Simple Summary

Feeding young pigs after weaning is challenging because their digestive systems are still developing, and high-fibre ingredients are often avoided despite their lower cost. Tiger nut is a promising alternative, but its fibre limits its use in pig diets. This study explored whether extrusion—a heat-based method used in commercial feed manufacturing—could improve tiger nut meal for nursery pigs. Based on the typical operating range of industrial single-screw extruders and the goal of optimising fibre modification while avoiding nutrient damage, the temperature range of 110–125 °C was chosen for testing. Testing four temperatures, it was found that processing at 110–120 °C made the fibre more functional—better at holding water and oil—and produced harder, more durable pellets, advantages for feed processing and transport. When fed to over 500 young pigs for three weeks, the extruded meal did not harm growth or digestion. Importantly, it also promoted healthier gut microbiota by encouraging beneficial bacteria while suppressing harmful ones. These findings suggest that extrusion can turn tiger nut meal into a valuable, cost-effective ingredient, offering farmers a practical way to reduce reliance on corn and soybean, while maintaining pig health and performance. The optimal range—110 to 120 °C—offers practical guidance for commercial feed mills.

## 1. Introduction

Pork represents a substantial portion of the global meat market, accounting for ~35% of the global meat supply [1]. Owing to Chinese dietary and consumption habits, China has become the world’s largest pork consumer. In 2025, the per capita pork consumption in China reached 29.5 kg, whereas the per capita beef consumption was only 5 kg [2]. This substantial market prospect has promoted pig farming in China. However, in response to fluctuations in feed ingredient supply, the search for diversified dietary formulations has attracted increasing attention [3].

*Cyperus esculentus* L., commonly known as tiger nut, produces hard tubers at the tips of its rhizomes. These tubers are round or oval in shape with a wrinkled brown epidermis. Historically, *C. esculentus* was used as food in ancient Egypt, and it continues to be consumed in regions such as Spain, Africa and China, where it is eaten raw, roasted or ground into powder for use in beverages [4]. *C. esculentus* demonstrates strong stress tolerance and possesses the capacity for windbreak and sand fixation. It can grow in arid, sandy and saline–alkali soils, thereby avoiding competition with staple food crops for arable land. The dry tuber yield can reach up to 7.5 tons per hectare [5]. The underground tubers of *C. esculentus* are rich in starch, fat, minerals and vitamins and contain bioactive compounds such as sterols, terpenoids, anthraquinones, flavonoids and organic acids. The oil extracted from the tubers is abundant in monounsaturated fatty acids, and its nutritional value is comparable to that of olive oil [6]. Consequently, it has emerged as a novel oil resource [7].

*C. esculentus* meal (CEM) is the by-product obtained following oil extraction from the tubers. CEM contains 25–35% starch, 5–10% crude protein and 9–13% crude fibre [8,9]. Notably, its protein exhibits an essential amino acid content (46.03%) superior to soybean meal (41.3%) [10], whereas its starch features low amylose content (13.9%) favouring gelatinisation [11]. As a by-product of oil extraction, CEM is a promising alternative feed ingredient [12].

However, its high fibre content, primarily comprising insoluble arabinoxylan [13], limits the utilisation of CEM in monogastric animals. Arabinoxylans increase digesta viscosity, reduce nutrient accessibility and promote endogenous nitrogen loss [14,15], ultimately impairing growth performance [9]. Extrusion technology provides a solution: high temperature (100–150 °C), pressure (3–8 MPa), and shear force disrupt fibre matrices, degrade antinutritional factors and enhance starch gelatinisation [16]. Critically, extrusion converts insoluble dietary fibre (IDF) to soluble fractions (SDF), thereby improving nutrient release [17]. Previous studies have demonstrated that incorporating appropriate amounts of extruded high-fibre ingredients, including corn straw and corn DDGS, into diets exerts no negative effects on growth performance in both finishing pigs and weaned piglets [18,19]. In addition, it has been demonstrated that including 10% extruded tiger nut meal in the diet enhances the growth performance of finishing pigs [20].

Although extrusion processing confers well-recognised advantages in feed manufacturing, including modification of protein conformation and improvement of lipid oxidative stability [21,22] the effects of varying extrusion temperatures on CEM have not been systematically elucidated. In particular, optimal processing temperatures that simultaneously balance fibre modification, nutrient preservation and pellet feed quality remain poorly defined. Previous studies have demonstrated that extrusion of whole tiger nut tubers at approximately 110 °C improves their physicochemical properties [23], and further investigations on defatted tiger nut flour have revealed that starch extraction yield decreases markedly when the extrusion temperature exceeds 140 °C [24], suggesting that excessive thermal treatment may exert detrimental effects on tiger nut-derived materials. However, because defatting fundamentally alters the chemical composition and processing behaviour of CEM compared with whole tubers, the optimal extrusion conditions established for whole tubers cannot be directly translated to CEM, necessitating systematic optimisation for this specific material. Accordingly, the present study selected 110–125 °C as the experimental temperature range and postulated that extrusion at an appropriate temperature would effectively improve the fibre physicochemical properties and pellet processing quality of CEM, while maintaining growth performance and promoting gut health in nursery pigs. To test this hypothesis, we systematically investigated the effects of graded extrusion temperatures on the fibre structure and functional properties of CEM, evaluated their subsequent impact on pellet feed quality, and further characterised the nutritional and physiological responses of nursery pigs fed diets containing extruded CEM. Collectively, this study not only elucidates the regulatory effects of extrusion temperature on the fibre characteristics and processing quality of CEM, but also provides practical extrusion parameters for its value-added utilisation and commercial application, offering a novel technological strategy for developing cost-effective functional feed ingredients and reducing dependence on corn and soybean meal in swine production.

## 2. Materials and Methods

### 2.1. Feed Processing and Dietary Treatment

*C. esculentus* meal containing 6.40% crude protein (CP), 10.00% crude fibre, and 8.50% moisture was obtained from a commercial source (Kunhua Biotechnology Co., Ltd., Anyang, China). Extrusion processing of CEM was performed using a single-screw extruder (die hole diameter 8–12 mm, TPH200C model, Muyang Group, Yangzhou, China) at four different extrusion temperatures (110 °C, 115 °C, 120 °C, and 125 °C). The moisture content of the mixture was adjusted to 21% and the screw speed was set at 320 r/min. The nutritional composition is presented in Table 1.

### 2.2. Fibre Analysis

#### 2.2.1. Determination of Fibre Content

The contents of neutral detergent fibre (NDF) and acid detergent fibre (ADF) were determined according to the Chinese national standards GB/T 20806 [25] and NY/T 1459 [26], respectively.

#### 2.2.2. Enzymatic Preparation of Fibre

The enzymes used in this experiment, including thermostable α-amylase (CAS 9001-19-8), neutral protease (CAS 9068-59-1) and amyloglucosidase (CAS 9032-08-0), were purchased from Shanghai Yuanye Bio-Technology Co., Ltd. (Shanghai, China) CEM fibre was extracted using a combination of these three enzymes, and the enzymatic hydrolysis process and parameters are described as follows. Exactly 25.0 g of CEM powder was placed into a 1000 mL conical flask, and distilled water was added at a solid-to-liquid ratio of 1:20. The pH was first adjusted to 6.1 ± 0.1 using 1 mol/L NaOH or HCl, followed by the addition of 1 mL thermostable α-amylase. The mixture was incubated in a water bath at 95 °C with shaking for 30 min. Then, the pH was adjusted to 7.5 ± 0.1, followed by the addition of 1 mL of neutral protease. Subsequently, the mixture was incubated in a constant-temperature shaking water bath at 40 °C for 2 h. Finally, the pH was adjusted to 4.6 ± 0.1 and 1 mL of amyloglucosidase was added with stirring. The mixture was incubated again in a constant-temperature shaking water bath at 40 °C for 30 min. Each enzymatic hydrolysis step was performed with the flask sealed by an aluminium foil cover. After enzymatic hydrolysis, the mixture was boiled for 5 min to inactivate the enzymes and then centrifuged at 4000 rpm for 10 min. Then, the precipitate was collected, washed twice with 75% ethanol and oven dried overnight at 50 °C to obtain the CEM fibre.

#### 2.2.3. Observation of Fibre Microstructure

The microstructure of the CEM fibre was observed using scanning electron microscopy (SEM, JSM-6390LV, JEOL Ltd., Tokyo, Japan). The dried sample was fixed onto an aluminium stub using double-sided adhesive tape and coated with a metal film using a vacuum-sputter coater to render it conductive. Sample images were captured at a magnification of 500× under an accelerating voltage of 10 kV.

#### 2.2.4. Physicochemical Properties of the Fibre

- Water-holding capacity

Approximately 0.5 g of fibre sample was placed in a 50 mL centrifuge tube, mixed with 20 mL of distilled water and allowed to stand at 22–25 °C for 2 h. The mixture was centrifuged at 4000 rpm for 10 min, and the supernatant was carefully removed. The weight of the precipitate was recorded. Water-holding capacity (WHC) was calculated as follows:

WHC (g/g) = (weight after water absorption − dry weight of sample)/dry weight of sample

- Oil-holding capacity

Approximately 0.5 g of fibre sample was placed in a 50-mL centrifuge tube, mixed with 20 mL of vegetable oil and allowed to stand at 22–25 °C for 2 h. The mixture was centrifuged at 4000 rpm for 10 min, and the precipitate was weighed. Oil-holding capacity (OHC) was calculated as follows:

OHC (g/g) = (weight after oil absorption − dry weight of sample)/dry weight of sample

- Swelling capacity

Exactly 1.0 g of sample was placed in a 25-mL graduated cylinder, and the initial volume was recorded. After the addition of 10 mL distilled water and thorough mixing, the mixture was allowed to stand at 22–25 °C for 12 h, and the final volume was recorded. Swelling capacity (SC) was calculated as follows:

SC (g/mL) = (final volume − initial volume)/dry weight of sample

### 2.3. Characteristics of Feed Pellets

Bulk density (g/L) was calculated as m1/V1 × 100, where m1 (g) is the weight of feed particles filled in a 1 L cylinder and levelled and V1 = 1 L. For pellet hardness, ~30 g of sample was taken by quartering, and 20 uniform pellets were randomly selected. Breaking force (N) was measured using a digital grain hardness tester, and the mean value was calculated from 30 replicates. The powder and disintegration rates were determined following the method of Chang et al. [27]. The degree of starch gelatinisation was analysed using a simplified enzymatic method widely adopted by the US feed industry, with reference to Xiong [28] for the determination of feed starch gelatinisation.

### 2.4. Nursery Pig Feeding Trial

The animal handling protocol (permit number: SYXK2020-0084) followed in the current study was approved by the Animal Care and Use Committee of the College of Animal Sciences and Technology, Huazhong Agricultural University, and complied with the National Research Council’s Guide for the Care and Use of Laboratory Animals. The Penglaixishipeng Farm in Yantai, Shandong, approved the animal studies.

A total of 528 Duroc × Landrace × Yorkshire pigs (initial BW = 13.87 kg) were randomly allocated to 6 treatments, resulting in 22 pigs per pen and 4 pens per treatment. The sample size was determined with reference to previous studies in swine nutrition that employed comparable designs [29]. Data were analysed with each pen serving as the experimental unit. The control group received a basal diet comprising corn–soybean meal. By replacing an equivalent amount of ordinary corn with 20% CEM and 20% extruded CEM (ECEM) processed at different temperatures (110 °C, 115 °C, 120 °C and 125 °C), five experimental diets were formulated. Diets were formulated according to the Nutrient Requirements of Swine. The diet formulation is presented in Table 2.

The experiment lasted for 21 days. Pigs were weighed after fasting at the beginning and end of the trial, with daily recordings of feed offered and residues to calculate average daily gain (ADG), average daily feed intake (ADFI) and feed conversion ratio (FCR).

The faecal consistency score was assessed daily and diarrhoea incidence was recorded according to the methods described by Yuan et al. [30] and Pu [31]. Fresh faecal samples were collected in the morning for 3 consecutive days before the end of the trial. Samples were placed in specimen bags and mixed with 5% hydrochloric acid at a ratio of 10:1 (faeces/HCl). Then, ~300 g of representative samples was transferred to plastic bags and stored at −20 °C for subsequent analysis. The calculation formulas are as follows:

Diarrhoea rate (%) = (Number of diarrhoea incidents during the trial/(Number of weaner pigs × Trial days)) × 100 Diarrhoea index = Sum of diarrhoea scores/(Number of weaner pigs × Trial days)

The apparent nutrient digestibility was determined using the endogenous indicator method with acid-insoluble ash as an inert marker. The apparent utilisation rate was calculated as follows:

Apparent utilisation rate (%) = [(target nutrient content in feed/indicator content in feed − target nutrient content in faeces/indicator content in faeces)/(target nutrient content in feed/indicator content in feed)] × 100

### 2.5. Blood Parameters

Prior to blood collection, the pigs were fasted for 16 h (with free access to water) to minimize the confounding effects of dietary intake on serum GLU and BUN levels. On the morning of the last day of the experiment, one pig from each replicate was randomly selected, and 5 mL of blood was collected from the jugular vein. After being left to stand for 1 h, the blood sample was centrifuged at 3000 rpm for 10 min to separate the serum, which was then stored at −20 °C until further analysis. Serum biochemical parameters were measured using a BK-280 automatic biochemical analyser (BIOBASE Group, Jinan, China). Serum antioxidant parameters were determined using corresponding kits purchased from Nanjing Jiancheng Bioengineering Institute (Nanjing, China).

### 2.6. Faecal Microbial Abundance

Fresh faecal samples were aseptically transferred into sterile cryotubes and immediately frozen, followed by preservation at −80 °C until further analysis. Total genomic DNA was extracted from faecal samples using the PowerSoil^®^ DNA Isolation Kit (MO BIO Laboratories, Carlsbad, CA, USA) following the manufacturer’s protocol. The V3–V4 hypervariable region of the bacterial 16S rRNA gene was amplified using the primers 338F (5′-ACTCCTACGGGAGGCAGCA-3′) and 806R (5′-GGACTACHVGGGTWTCTAAT-3′). Sequencing was performed on an Illumina NovaSeq 6000 platform (Illumina, San Diego, CA, USA) by Biomarker Technologies Co., Ltd. (Beijing, China). Bioinformatics analysis was conducted using the BMK Cloud platform (Biomarker Technologies, Beijing, China).

### 2.7. Statistical Analysis

The experimental data were processed using Excel 2019 and analysed using SPSS 24. Normality was evaluated using the Shapiro–Wilk test. For data with homogeneous variance, one-way analysis of variance (ANOVA) followed by Duncan’s test was conducted; for data with heterogeneous variance, Welch’s ANOVA with Tamhane’s T2 post hoc test was applied. The results are expressed as mean ± SD, with significance thresholds set at *p* < 0.05 (significant), *p* < 0.01 (highly significant) and 0.05 < *p* < 0.1 indicating a trend.

## 3. Results

### 3.1. Effects of Different Extrusion Temperatures on the Physicochemical Properties and Microstructure of C. esculentus Meal Fibre

As shown in Table 3, extrusion temperature significantly affected the fibre content of CEM. The NDF content markedly decreased after extrusion (*p* < 0.01), reaching its lowest level at 120 °C. The ADF content exhibited no significant difference between CEM and 115-°C ECEM but was significantly reduced in the other treatment groups. Among these, 110-°C ECEM exhibited the lowest ADF content, with significant differences compared with CEM, 115-°C ECEM (*p* < 0.001) and 120-°C and 125-°C ECEM (*p* < 0.01).

Extrusion treatment increased the WHC of CEM fibre. As extrusion temperature rose, WHC first increased and then decreased. The WHC of ECEM fibre at 120 °C was significantly higher than that of CEM fibre (*p* < 0.05). The OHC also first increased and then decreased as extrusion temperature rose, reaching its maximum value in the ECEM fibre treated at 110 °C. The OHC of ECEM fibre at 125 °C was significantly lower than that of CEM fibre (*p* < 0.05).

Extrusion treatment significantly increased the SC of CEM fibre. Compared with ECEM fibre at 110 °C, the SC values of ECEM fibres at 115 °C, 120 °C and 125 °C were significantly higher (*p* < 0.001).

**Figure 1:**
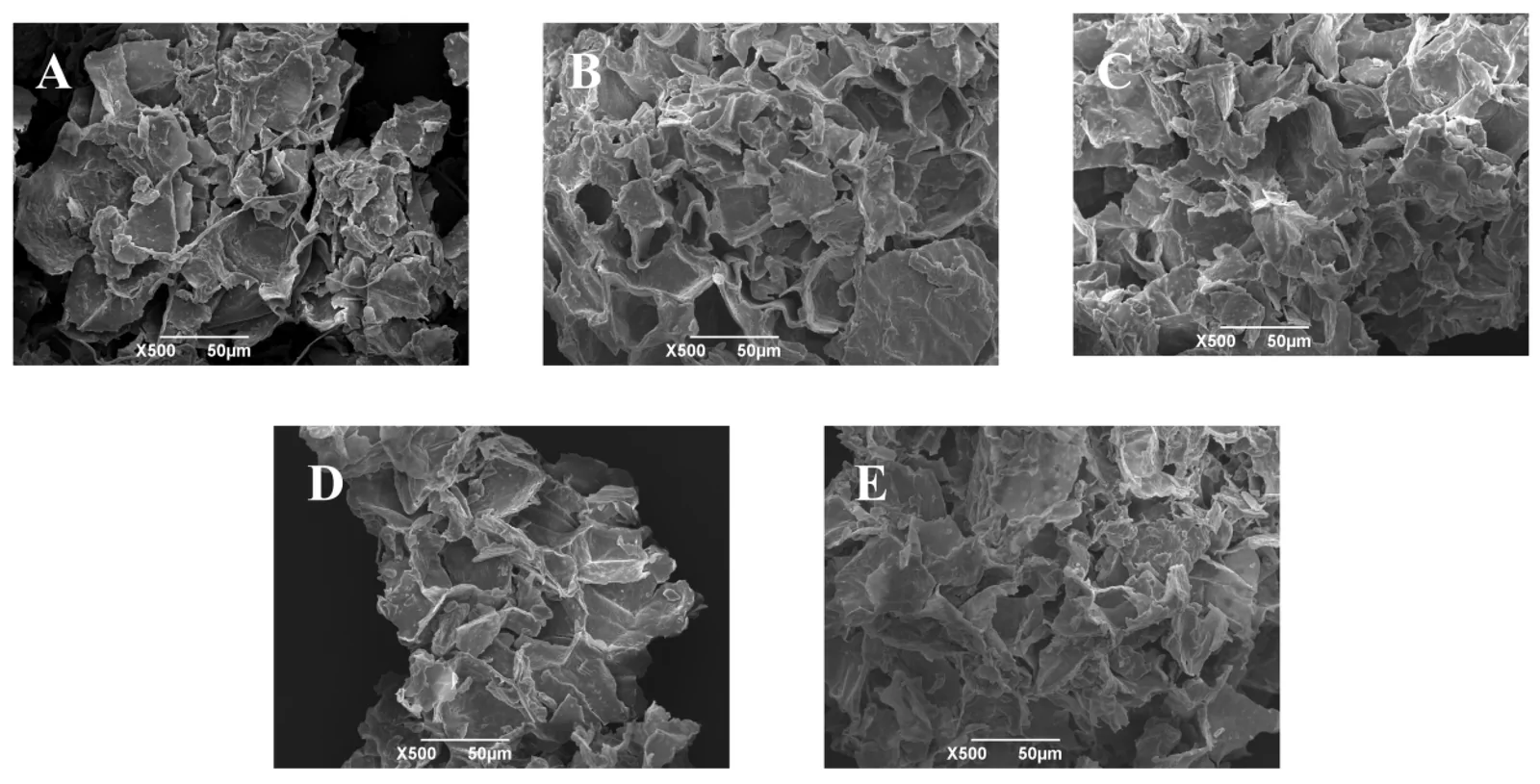
Scanning electron microscope image of fibre (×500 times) before and after CEM extrusion. Notes: (**A**) CEM fibre; (**B**) 110-°C ECEM fibre; (**C**) 115-°C ECEM fibre; (**D**) 120-°C ECEM fibre; (**E**) 125-°C ECEM fibre.

Figure 1 presents SEM images (500× magnification) of fibres from unexpanded CEM and CEM expanded at four different temperatures. The fibre structure of unexpanded CEM appears dense and compact with a smooth surface. The fibre structures of ECEM at 110 °C, 115 °C, 120 °C and 125 °C are loose and porous, with wrinkled surfaces, numerous pores and cracks, and an increased specific surface area.

### 3.2. Effects of Different Extrusion Temperatures on Pellet Feed Quality

As presented in Table 4, extrusion temperature significantly influenced pellet hardness, bulk density, powder rate, disintegration rate and starch gelatinisation degree. The CEM group and all ECEM groups exhibited significantly greater pellet hardness than the control group (*p* < 0.001), and the 115-°C ECEM group showed significantly higher hardness than the 110-°C, 120-°C and 125-°C ECEM groups. Compared with the control and CEM groups, all ECEM groups showed significantly lower bulk density (*p* < 0.001), and the bulk density of the 110-°C, 115-°C and 120-°C ECEM groups was significantly lower than that of the 125-°C ECEM group (*p* < 0.001). The powder rate and disintegration rate of all ECEM groups were significantly lower than those of the control and CEM groups (*p* < 0.001), with the 115-°C ECEM group having the lowest powder rate and the 120-°C ECEM group showing the lowest disintegration rate. No significant differences in starch gelatinisation degree were observed among the groups.

### 3.3. Effects of Extruded C. esculentus Meal on the Growth Performance of Nursery Pigs

The effects of different extrusion temperatures of CEM on the growth performance of nursery pigs are presented in Table 5. As shown in the table, feeding CEM and ECEM exerted no significant effects on the final body weight, ADG, ADFI or FCR of the nursery pigs.

### 3.4. Effect of Extruded C. esculentus Meal on Diarrhoea Incidence in Nursery Pigs

The effects of different extrusion temperatures of CEM on diarrhoea indicators in nursery pigs are presented in Table 6. As shown in the table, no significant differences were observed among groups for either diarrhoea incidence or diarrhoea index during any of the observed periods (days 1–7, 8–14, 15–21 or 1–21).

### 3.5. Effects of Extruded C. esculentus Meal on Nutrient Digestibility in Nursery Pigs

The effects of different expansion temperatures of CEM on nutrient digestibility in nursery pigs are presented in Table 7. As shown in the table, there were highly significant differences in dry matter (DM) digestibility among the groups (*p* < 0.01). No significant differences were observed in CP digestibility among the groups, although the 115-°C ECEM group had significantly lower CP digestibility than the 120-°C ECEM group (*p* < 0.05). Ether extract (EE) digestibility significantly differed among the groups (*p* < 0.001). Ash digestibility significantly varied among the groups (*p* < 0.05).

### 3.6. Effects of Extruded C. esculentus Meal on Serum Biochemical Parameters in Nursery Pigs

As shown in Table 8, the 125-°C ECEM group exhibited significantly higher total protein (TP) content than those of the CEM, 115-°C ECEM and 120-°C ECEM groups (*p* < 0.05), whereas no significant differences were observed between the CEM, ECEM and control groups. The alanine aminotransferase (ALT) levels in both the CEM and 120-°C ECEM groups were significantly lower than those in the control and 125-°C ECEM groups (*p* < 0.05). For the alkaline phosphatase (ALP) content, the CEM, 110-°C, 115-°C and 125-°C ECEM groups showed significantly lower values than the control and 120-°C ECEM groups (*p* < 0.001). No significant differences were observed among groups in glucose (GLU) levels (*p* = 0.067) or blood urea nitrogen (BUN) levels (*p* = 0.062). No significant effects of CEM substitution or ECEM substitution were observed on other serum biochemical parameters.

### 3.7. Effect of Different Extrusion Temperatures of CEM on Serum Antioxidant Indices in Nursery Pigs

As shown in Table 9, the T-AOC level in the 125-°C ECEM group was significantly lower than that in all of the other groups (*p* < 0.001). The MDA content in all ECEM groups was significantly higher than that in the control and CEM groups (*p* < 0.001). Regarding GSH-Px activity, the 125-°C ECEM group exhibited significantly lower levels than the control, 110-°C ECEM, and 120-°C ECEM groups (*p* < 0.001). No significant differences were observed in SOD activity among the groups. The CAT activity in the CEM and 110-°C ECEM groups was significantly higher than that in the control, 120-°C ECEM and 125-°C ECEM groups (*p* < 0.01).

### 3.8. Effects of Extruded C. esculentus Meal on Faecal Microbiota in Nursery Pigs

#### 3.8.1. Effects of Extruded *C. esculentus* Meal on Faecal Microbial Alpha Diversity in Nursery Pigs

As presented in Table 10, dietary supplementation with CEM and ECEM had no significant effects on the Chao1, ACE, Simpson or Shannon index of faecal microbiota in nursery pigs. The Shannon index in the ECEM groups was higher than that in the control group, although the difference did not reach statistical significance (*p* = 0.057). The alpha diversity indices in the 110-°C ECEM group were significantly higher than those in the other groups. The Simpson index in all groups was close to 1.

#### 3.8.2. Effects of Extruded *C. esculentus* Meal on Taxonomic Analysis of Faecal Microbiota in Nursery Pigs

As shown in Table 11, at the phylum level, the four phyla with the highest relative abundance in the faecal microbiota of nursery pigs were Firmicutes, Bacteroidota, Spirochaetota and Desulfobacterota, accounting for >96%. Firmicutes exhibited the highest relative abundance among the six groups, followed by Bacteroidota; these two phyla together accounted for >85%, making them the dominant phyla. No significant differences were observed in the relative abundance of Firmicutes, Bacteroidota, Spirochaetota or Desulfobacterota among the six groups. Bacteroidota abundance was numerically higher in the ECEM groups, but the difference was not significant (*p* = 0.086).

As shown in Table 12, at the genus level, the relative abundance of faecal microbiota in each group was as follows: Porphyromonadaceae exhibited the highest relative abundance, followed by Prevotellaceae, Lachnospiraceae, Lactobacillus, Treponema and Bacilli. However, there were no significant differences in other genera among the groups.

#### 3.8.3. Analysis of Differences in Faecal Microbiota Among the Groups of Nursery Pigs

**Figure 2:**
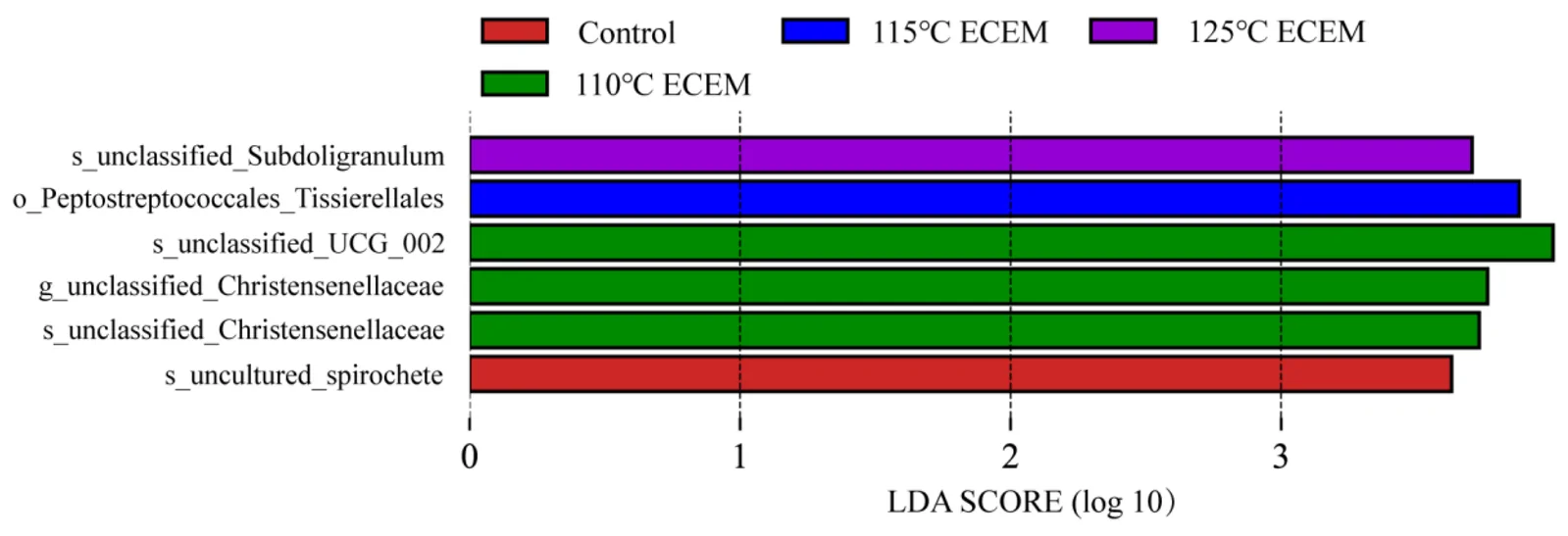
Histogram of LDA scores.

As shown in Figure 2, no marker species with an LDA score of >3.5 were identified in the CEM or the 120-°C ECEM group. The marker species in the control group was Spirochete. In the 110-°C ECEM group, the marker species were UCG_002 and Christensenellaceae, with UCG_002 having the highest LDA value in this group. The marker species in the 115-°C ECEM group was Peptostreptococcales_Tissierellales, and the marker species in the 125-°C ECEM group was Subdoligranulum.

## 4. Discussion

CEM contains a high proportion of crude fibre, mainly comprising xylan and arabinose, which increases digesta viscosity in the intestine, hinders the digestion and absorption of nutrients, and impairs animal growth [13]. Numerous studies have reported that extrusion can break the glycosidic and hydrogen bonds of dietary fibre (DF) in raw materials, converting it into an easily digestible soluble dietary fibre (SDF) [32], and reduces NDF and ADF contents [33,34,35]. The results of the present study also show that extrusion reduces the NDF and ADF contents in CEM, effectively improving the quality of feed ingredients.

Extrusion also modifies fibre physical structure via high temperature, pressure, and shear forces, creating a honeycomb-like porous surface with reduced particle size and facilitating water and oil entrapment [36,37]. This enhances water-holding capacity (WHC), oil-holding capacity (OHC), and swelling capacity (SC) [38], which are key hydration properties that increase digesta viscosity, enhance satiety, and benefit intestinal motility [39,40]. OHC reflects the lipid-adsorption capacity of DF, and its increase contributes to reduced fat absorption and improved laxation [41]. Qiao et al. found that extruded sweet potato residue developed a coarser and more porous structure with significantly enhanced hydration properties [42]; scanning electron microscopy (SEM) images in this study also confirmed that extruded CEM exhibited a folded, multi-layered, and porous morphology with increased specific surface area, correlating with the observed improvements in WHC and SC. Notably, OHC peaked at 110 °C but dropped below untreated levels at 125 °C, suggesting that moderate extrusion opens the fibre structure and enhances lipid adsorption, whereas excessive temperature may damage the capillary matrix and embed functional groups, thereby reducing hydration capacity and OHC [43]. Overall, moderate extrusion effectively enhances the functional quality of CEM as a feed ingredient, laying a foundation for its improved processing behaviour and nutrient availability.

Studies have demonstrated that the raw material composition of feed formulations influences pellet quality [44]. Pellet hardness is a key indicator for evaluating pig feed quality, which directly influences palatability and feed intake [45]. Nursery pigs have a relatively weak digestive capacity; overly hard pellets reduce nutrient digestibility, whereas overly soft ones increase fines content and disintegration rate, thereby resulting in feed waste and economic losses [46]. Changes in pellet hardness involve the synergistic effects of multiple components. First, the degree of starch gelatinization is a key factor: gelatinization increases viscosity, with amylose contributing to hardness and amylopectin improving binding forces, thereby enhancing pellet durability [47,48,49]. Second, protein denaturation under heat unfolds peptide chains, increasing viscosity and facilitating pellet formation [50,51]. Furthermore, pellet hardness is closely related to dietary fibre (DF) level. An appropriate fibre content can increase feed viscosity and reduce porosity, thereby improving pellet stability. However, excessive fibre can hinder pellet formation, whereas extrusion can partially convert insoluble fiber into soluble forms to alleviate this issue [52]. In this study, the pellet hardness in both the CEM and ECEM groups was significantly higher than that in the control group, supporting the existing conclusion that “higher fibre levels lead to greater hardness.” Notably, as the extrusion temperature of ECEM increased, both pellet hardness and starch gelatinization degree showed a trend of first increasing and then decreasing, suggesting that extrusion temperature may affect hardness by regulating gelatinization, and that excessively high temperatures may instead disrupt system stability.

ECEM treatment optimized pellet hardness while simultaneously reducing the fines percentage by approximately 40% when compared with the control and CEM groups, an effect attributable to the enhanced binding and durability of pellets resulting from extrusion-induced starch gelatinization [47]. However, this improvement in physical quality did not translate into a significant enhancement in growth performance, which is consistent with the finding of Ge et al. [46] that pellet hardness is not directly correlated with growth performance. A possible explanation is that the ad libitum feeding regimen adopted throughout the trial allowed pigs to self-regulate their feeding behaviour according to individual preferences, thereby buffering the actual impact of pellet physical properties on single-meal intake and digestive efficiency, and thus limiting the transmission of quality differences to growth performance. In summary, the ECEM treatment optimized pellet hardness and reduced fines percentage through extrusion temperature-modulated starch gelatinization, conferring practical value in reducing feed wastage. It should be noted, however, that the present study did not include behavioural observations or preference tests, which precludes direct assessment of how pellet quality variation affects feeding preference and palatability sensitivity in weaned piglets. Future research should combine comparative designs of restricted versus ad libitum feeding, along with behavioural tracking or two-choice preference assays, to more comprehensively elucidate the pathways through which pellet physical properties influence feeding behaviour and production performance in piglets.

Pigs fed extruded CEM (ECEM) exhibited a general trend of nutrient digestibility first increasing and then decreasing with increasing extrusion temperature, with the 120 °C treatment group showing the closest apparent total tract digestibility (ATTD) of dry matter (DM), crude protein (CP), and crude ash to that of the corn-soybean meal control group. In contrast, the ATTD of DM and ether extract (EE) in the untreated CEM group was significantly lower than that in the control group, with a decreasing trend in ash digestibility, consistent with the findings of Shi et al. [9]. This indicates that under optimized conditions, extrusion can effectively improve conventional nutrient utilization. The improvement in digestibility following extrusion could be attributed to the reduced NDF and ADF contents, which alleviated their physical interference with digestive enzymes and nutrients [53]. Similarly, Xie et al. reported that extrusion processing improved the digestibility of energy, dry matter, and fibre fractions in brewery by-products for growing pigs [54]. However, the variability in digestibility among ECEM groups highlighted the delicate balance inherent in extrusion processing—optimal conditions enhanced nutrient utilization, whereas excessive temperatures might induce Maillard reactions or excessive denaturation, thereby reducing the digestibility of protein and other nutrients [55].

Despite significant differences in nutrient digestibility among treatment groups, no significant changes in growth performance were observed in pigs fed the control, CEM, or ECEM diets. These results indicate that both untreated and extruded CEM can replace 20% of corn in weaned piglet diets without compromising growth performance, which is consistent with the findings of Shi et al. [9], who used a 10% replacement level. The discrepancy in replacement levels between the two studies may be attributed to differences in the geographical origin and nutritional composition of the CEM used. Likewise, the lack of growth response to extrusion, despite improved digestibility in some ECEM groups, is consistent with the reports of Liermann et al. [56] and Ren et al. [57]. This discrepancy may be attributable to the relatively short experimental period or to weaning and regrouping-related stress, which might have masked the potential performance advantages of extrusion processing and extrusion temperatures. In summary, although certain differences in pellet quality and nutrient digestibility were observed among treatment groups, neither the CEM nor the ECEM groups adversely affected the growth performance of nursery pigs when replacing corn, confirming the potential of CEM as an unconventional feed ingredient. However, it should be noted that the experimental design included only one control group, and that the proportion of corn replacement differed markedly between the control and treatment groups (0 vs. 20%). Consequently, the observed differences in pellet quality and digestibility may have been partially confounded by variations in dietary formulation, making it difficult to attribute these effects solely to ingredient substitution. Future studies should incorporate multiple gradient replacement levels with isonitrogenous and isoenergetic controls, or adopt a 2 × 2 factorial design (replacement ratio × extrusion treatment), to more clearly distinguish the respective contributions of ingredient substitution and extrusion processing.

Tiger nut is rich in flavonoids and phenolic acids, which have been shown to increase serum T-AOC, CAT, and SOD activities in growing pigs, thereby significantly enhancing antioxidant capacity [58,59,60]. However, in the present study, CEM feeding did not significantly affect these antioxidant indices, suggesting that the antioxidant effects of tiger nut may be condition-dependent, with their in vivo efficacy modulated by factors such as inclusion level, processing method, or physiological state. The increasing trend in GLU levels aligns with the expectation that extrusion enhances starch gelatinization and thus accelerates glucose release [61]. ALP levels were generally reduced across treatment groups, except for the 120 °C ECEM group, which may be related to reduced feed intake and impaired intestinal absorption associated with diarrhoea. Notably, the trend of ALT activity first decreasing and then recovering with increasing extrusion temperature suggests that antioxidant and anti-inflammatory compounds in tiger nut may alleviate hepatic oxidative stress and exert hepatoprotective effects [62], although excessive heat may attenuate this protection. Furthermore, the elevated MDA levels in the 120 °C and 125 °C ECEM groups, which compromised antioxidant reserves and led to accumulation of lipid peroxidation products, may be attributed to thermal degradation of endogenous antioxidants (e.g., vitamin E) in corn during high-temperature extrusion [63]. This interpretation aligns with reports that heat-processed feed ingredients can induce systemic oxidative stress and deplete endogenous antioxidant reserves [64]. Collectively, the serum biochemical and antioxidant responses indicate that extrusion temperature exerts a double-edged effect: moderate temperatures may preserve or even enhance the bioactive potential of CEM, whereas excessive temperatures carry the risk of thermolabile antioxidant degradation. This trade-off warrants careful consideration when optimizing extrusion conditions in practical applications.

Weaned piglets have incomplete intestinal development and are highly susceptible to stress caused by external environmental changes and dietary transitions, leading to diarrhoea, intestinal barrier damage, and alterations in gut microbiota structure [65]. Firmicutes and Bacteroidetes are the two dominant phyla in pig faeces [66] and are closely associated with starch and fibre digestion and metabolism [67]. In this study, the abundance of Bacteroidetes in pigs fed CEM and ECEM exhibited an increasing trend, which may be related to the extrusion-induced enhancement of starch gelatinization that improves starch digestibility. Concurrently, the increased abundance of bacterial groups proficient in degrading complex polysaccharides—Bacteroidetes and Ruminococcaceae—indicates that extruded CEM provides more fermentable carbohydrates and supports energy harvest [68]. At the genus level, Porphyromonas has been associated with hepatic TNF-α expression and serum ALP levels [69], which aligns with the magnitude of ALP changes observed in this trial. Notably, potential pathogenic taxa, including Spirochaetaceae and Streptococcaceae, were suppressed across all ECEM groups, suggesting a healthier microbial ecosystem.

LEfSe analysis further identified the biomarkers of each treatment group. The biomarker of the control group was Spirochaeta, a genus that contains pathogenic species [70] and may induce diarrhoea in pigs. Ruminococcaceae UCG_002 and Christensenellaceae were the biomarkers in the 110 °C ECEM group. Ruminococcaceae UCG_002 is potentially involved in carbohydrate digestion, and Christensenellaceae can utilize sugars to produce volatile fatty acids; both taxa are related to glucose metabolism [71,72]. This reflects the extrusion-induced enhancement of starch gelatinization and reduction in IDF, which collectively promote carbohydrate utilization. Peptostreptococcus was the biomarker in the 115 °C group, which promotes cholesterol synthesis [73]. Massilibacterium was the biomarker in the 125 °C group, which can ferment carbohydrates to produce short-chain fatty acids and succinic acid [74]. The above microbial shifts indicate that ECEM upregulates gut microbiota associated with glucose and lipid metabolism, thereby enhancing energy utilization efficiency. Similar patterns of microbiota remodelling have also been reported in studies on functional feed additives (e.g., yeast-derived postbiotics) and unconventional feed ingredients (e.g., vinegar residue)—both of which observed enrichment of carbohydrate-degrading microbiota and proliferation of short-chain fatty acid-producing bacteria, accompanied by improved energy utilization efficiency [75,76]. The enrichment of Bacteroidetes and Ruminococcaceae observed in this study is consistent with the above trends, further suggesting that ECEM, as a sustainable feed alternative, holds application potential in improving intestinal health and energy metabolism in weaned piglets. These microbial shifts, together with the optimised fibre configuration and improved pellet physical properties, collectively indicate that the nutritional enhancement of CEM by extrusion is not merely a change in chemical composition, but a multi-faceted, integrated improvement.

## 5. Conclusions

In conclusion, extrusion at 110–120 °C improves the functional properties of CEM and enhances pellet quality, nutrient digestibility, and gut microbiota, without compromising growth performance. These findings support the use of extruded CEM as a functional alternative ingredient in nursery pig diets, offering a practical strategy to reduce reliance on corn and soybean meal and promote the feed utilisation of fibrous by-products in commercial pig production.

## Figures and Tables

**Table 1:** Nutrition composition and content of antinutritional factors of CEM and ECEM (%, air-dry basis).

	CEM	110-°C ECEM	115-°C ECEM	120-°C ECEM	125-°C ECEM
Dry matter	91.50	93.50	93.70	92.90	94.00
Crude protein	6.40	6.90	6.83	6.83	7.02
Ether extract	2.20	1.30	1.50	1.30	1.40
Ash	3.90	4.40	4.40	4.40	4.70
Crude fibre	10.00	7.60	8.30	7.70	6.80
Neutral detergent fibre	20.10	16.90	20.50	17.80	16.40
Acid detergent fibre	11.00	8.80	10.70	9.60	8.70
Starch gelatinisation degree	13.00	98.00	95.70	84.70	95.80
Starch	35.24	36.28	36.77	36.91	36.95
Fructose	1.30	0.42	0.59	0.56	0.56
Glucose	1.30	0.34	0.42	0.43	0.38
Sucrose	16.60	18.00	17.10	16.20	15.20
Met	0.14	0.17	0.16	0.15	0.19
Cys	0.01	0.01	0.01	0.02	0.01
Phe	0.17	0.18	0.16	0.16	0.16
Ala	0.33	0.34	0.34	0.36	0.32

*Cyperus esculentus* meal was extruded at temperatures of 110 °C, 115 °C, 120 °C and 125 °C, respectively. After processing, the contents of nutrients and antinutritional factors were determined. All amino acid values are expressed as total amino acids determined by near-infrared reflectance spectroscopy (NIRS).

**Table 2:** Ingredient and chemical composition (g/kg as fed) of the basal diets.

Ingredients (%)	Control Group	Test Group
Corn	60.81	40.90
CEM/ECEM	0.00	20.00
Soybean meal	15.00	13.70
Expanded soybean	6.00	6.00
Flour	6.00	6.00
Fermented soybean meal	5.00	5.00
Soya bean oil	1.80	1.90
Calcium hydrogen phosphate I	1.71	1.66
L-threonine	0.21	1.30
L-lysine hydrochloride	0.56	0.61
Sodium chloride	0.36	0.36
Montmorillonite	0.30	0.30
Soybean phospholipid oil powder	0.50	0.50
Stone powder	0.28	0.27
DL-methionine	0.24	0.26
L-tryptophan	0.05	0.06
Phytase	0.02	0.02
Premix ^1^	1.17	1.17
Total	100.00	100.00
Nutritional composition ^2^		
CP	17.98	18.00
CF	2.37	2.74
EE	5.62	5.48
Ash	4.84	5.26
Ca	0.63	0.63
P-total	0.63	0.66
Lysine	1.32	1.32

^1^ Premix provides the following per kilogram of diet: VA2 104 IU, VD 300 IU, VE 6.66 IU, VK3 0.83 IU, VB2 1.53 mg, VB6 0.9 mg, VB12 6 μg, pantothenic acid 5.0 mg, niacin 10.1 mg, biotin 0.02 mg, Cu 33.33 mg, Fe 30 mg, Mn 13.33 mg, Zn 26.67 mg, I 0.28 mg, Se 0.08 mg. ^2^ The nutritional compositions are calculated values.

**Table 3:** Effects of different extrusion temperatures on the physicochemical properties of *Cyperus esculentus* meal fibre.

	Treatment					*p* Value
	CEM	110-°C ECEM	115-°C ECEM	120-°C ECEM	125-°C ECEM	
NDF (%)	57.96 ± 0.89 a	51.68 ± 1.87 b	51.60 ± 2.82 b	50.89 ± 1.03 b	51.50 ± 2.23 b	0.006
ADF (%)	13.10 ± 0.34 a	10.89 ± 0.07 c	13.18 ± 0.46 a	11.64 ± 0.15 b	11.97 ± 0.40 b	<0.001
Water-holding capacity	2.98 ± 0.26 b	3.72 ± 0.13 ab	4.13 ± 0.03 a	4.23 ± 0.27 a	3.62 ± 0.76 ab	0.019
Oil-holding capacity	4.70 ± 0.30 bc	5.92 ± 0.13 a	4.75 ± 0.11 bc	4.85 ± 0.21 b	4.50 ± 0.03 c	<0.001
Swelling capacity	0.79 ± 0.21 d	1.60 ± 0.11 c	3.85 ± 0.21 ab	3.69 ± 0.16 b	4.05 ± 0.07 a	<0.001

Values are expressed as means ± standard deviation (SD) of determinations. a–d Within a row, means without a common superscript letter significantly differ (*p* < 0.05). CEM, unextruded *Cyperus esculentus* meal; ECEM, extruded *Cyperus esculentus* meal; NDF, neutral detergent fibre; ADF, acid detergent fibre.

**Table 4:** Effects of different extrusion temperatures on pellet feed quality.

	Treatment						*p* Value
	Corn–Soybean Meal Type	CEM	110-°C ECEM	115-°C ECEM	120-°C ECEM	125-°C ECEM	
Bulk density (g/L)	657.3 ± 0.72 a	652.53 ± 4.04 a	619.37 ± 4.54 c	617.13 ± 1.89 c	618.83 ± 2.74 c	635.67 ± 4.73 b	<0.001
Fines content (%)	5.42 ± 0.53 a	5.4 ± 0.88 a	2.16 ± 0.41 b	1.93 ± 0.15 b	2.76 ± 0.27 b	2.33 ± 0.52 b	<0.001
Pellet durability index (%)	15.3 ± 1.47 a	16.65 ± 1.6 a	7.04 ± 0.33 b	7.02 ± 0.28 b	6.85 ± 1.02 b	7.58 ± 0.9 b	<0.001
Hardness (N)	29.47 ± 5.98 c	45.83 ± 12.04 b	43.36 ± 8.89 b	52.67 ± 12.96 a	42.9 ± 10.97 b	42.99 ± 9.15 b	<0.001
Gelatinisation degree (%)	41.63 ± 4.16	46.01 ± 6.71	46.74 ± 6.7	49.77 ± 9.19	43.99 ± 2.83	38.38 ± 3.13	0.295

Values are expressed as means ± standard deviation (SD) of determinations. Pellet samples from each group were obtained using the quartering method and used for the determination of pellet quality characteristics. a–c Within a row, means without a common superscript letter significantly differ (*p* < 0.05).

**Table 5:** Effects of different extrusion temperatures of CEM on the growth performance of nursery pigs.

	Treatment						*p* Value
	Control	CEM	110-°C ECEM	115-°C ECEM	120-°C ECEM	125-°C ECEM	
IBW (kg)	13.83 ± 1.25	13.82 ± 2.33	13.70 ± 2.25	13.89 ± 1.65	13.74 ± 1.56	13.78 ± 1.84	0.999
FBW (kg)	22.30 ± 2.68	22.17 ± 3.95	22.17 ± 3.42	22.45 ± 2.82	22.34 ± 1.92	22.70 ± 3.32	0.999
ADG (g)	403.19 ± 69.50	397.86 ± 78.73	403.03 ± 56.55	407.79 ± 60.88	409.50 ± 21.84	424.62 ± 71.61	0.993
ADFI (g)	746.23 ± 103.40	741.28 ± 135.31	749.54 ± 94.72	747.68 ± 107.47	734.27 ± 43.09	780.71 ± 121.58	0.992
F/G	1.86 ± 0.10	1.87 ± 0.13	1.86 ± 0.10	1.84 ± 0.11	1.79 ± 0.10	1.84 ± 0.05	0.913

Values are expressed as means ± standard deviation (SD) of determinations. A total of 528 DLY crossbred pigs (initial BW = 13.87 kg) were randomly assigned to 6 dietary treatments, with 22 pigs per pen and 4 replicate pens per treatment. A corn–soybean meal basal diet served as the control. Experimental diets were formulated by substituting 20% ordinary corn with 20% *Cyperus esculentus* meal (CEM) or 20% extruded CEM (ECEM) processed at 110 °C, 115 °C, 120 °C and 125 °C, respectively. IBW, initial body eight; FBW, final body weight; ADG, average daily gain; ADFI, average daily feed intake; F/G, feed to gain ratio.

**Table 6:** Effects of different extrusion temperatures of CEM on diarrhoea indicators in nursery pigs.

	Treatment						*p* Value
	Control	CEM	110-°C ECEM	115-°C ECEM	120-°C ECEM	125-°C ECEM	
1–7 d							
Diarrhoea rate	5.21 ± 3.99	5.32 ± 2.94	10.30 ± 3.62	2.60 ± 1.68	6.36 ± 4.51	6.66 ± 3.24	0.190
Diarrhoea index	0.09 ± 0.06	0.09 ± 0.05	0.18 ± 0.07	0.05 ± 0.03	0.10 ± 0.08	0.10 ± 0.06	0.158
8–14 d							
Diarrhoea rate	4.41 ± 2.64	3.54 ± 2.41	5.59 ± 3.03	2.93 ± 0.84	4.28 ± 2.55	5.49 ± 4.61	0.755
Diarrhoea index	0.09 ± 0.06	0.08 ± 0.04	0.13 ± 0.06	0.05 ± 0.02	0.11 ± 0.05	0.14 ± 0.11	0.364
15–21 d							
Diarrhoea rate	2.16 ± 1.82	2.89 ± 2.39	2.13 ± 1.69	2.84 ± 1.53	2.00 ± 1.42	2.51 ± 1.30	0.960
Diarrhoea index	0.05 ± 0.04	0.07 ± 0.06	0.05 ± 0.04	0.06 ± 0.03	0.05 ± 0.04	0.06 ± 0.04	0.980
1–21 d							
Diarrhoea rate	4.00 ± 1.72	3.88 ± 2.20	6.04 ± 2.60	2.78 ± 0.80	4.22 ± 2.42	4.90 ± 2.66	0.438
Diarrhoea index	0.08 ± 0.03	0.08 ± 0.04	0.12 ± 0.05	0.06 ± 0.02	0.08 ± 0.05	0.10 ± 0.05	0.438

Values are expressed as means ± standard deviation (SD) of determinations. Pens were inspected every morning to record the number of diarrhoea incidents and score faecal consistency.

**Table 7:** Effect of different extrusion temperatures of CEM on nutrient digestibility in nursery pigs (%).

	Treatment						*p* Value
	Control	CEM	110-°C ECEM	115-°C ECEM	120-°C ECEM	125-°C ECEM	
DM	88.81 ± 2.35 a	84.90 ± 2.12 bc	83.10 ± 0.86 c	83.45 ± 1.42 c	86.33 ± 0.28 ab	85.17 ± 1.97 bc	0.002
CP	84.30 ± 3.64	81.88 ± 2.88	80.69 ± 1.88	77.88 ± 3.33	82.43 ± 0.84	79.45 ± 2.95	0.053
EE	92.06 ± 1.56 a	89.08 ± 1.85 bc	87.22 ± 0.85 c	86.85 ± 1.08 d	89.64 ± 1.43 b	90.05 ± 1.24 ab	<0.001
Ash	54.44 ± 7.87 a	47.97 ± 5.78 ab	42.96 ± 1.52 b	44.47 ± 4.63 b	54.04 ± 0.47 a	50.46 ± 5.41 ab	0.017

Values are expressed as means ± standard deviation (SD) of determinations. Four samples of each group were used to determine the nutrient digestibility of DM and CP (%). DM, dry matter; CP, crude protein; EE, ether extract. a–d Within a row, means without a common superscript letter significantly differ (*p* < 0.05).

**Table 8:** Effect of different extrusion temperatures of CEM on serum biochemical indices in nursery pigs.

	Treatment						*p* Value
	Control	CEM	110-°C ECEM	115-°C ECEM	120-°C ECEM	125-°C ECEM	
TP (g/L)	53.75 ± 3.32 ab	50.75 ± 4.90 b	53.72 ± 7.69 ab	46.36 ± 4.60 b	47.10 ± 3.75 b	61.39 ± 9.49 a	0.027
GLU (mmol/L)	5.38 ± 0.41	5.76 ± 1.10	5.81 ± 0.43	5.79 ± 0.88	6.21 ± 0.65	7.05 ± 0.62	0.067
ALT (U/L)	37.80 ± 5.77 a	26.61 ± 3.57 b	30.43 ± 7.74 ab	32.30 ± 3.84 ab	28.16 ± 8.38 b	38.81 ± 4.41 a	0.045
ALP (U/L)	267.20 ± 45.90 a	147.94 ± 32.21 b	190.42 ± 16.82 b	168.12 ± 37.19 b	261.80 ± 31.28 a	175.80 ± 15.87 b	<0.001
BUN (mmol/L)	3.68 ± 1.59	2.65 ± 0.67	2.30 ± 0.28	1.96 ± 0.29	2.08 ± 0.83	3.41 ± 0.97	0.062

Values are expressed as means ± standard deviation (SD) of determinations. One pig was randomly selected from each replicate for blood sampling, with four pigs per treatment in total. a–b Within a row, means without a common superscript letter significantly differ (*p* < 0.05). TP, total protein; GLU, glucose; ALT, alanine aminotransferase; ALP, alkaline phosphatase; BUN, blood urea nitrogen.

**Table 9:** Effect of different extrusion temperatures of CEM on serum antioxidant indices in nursery pigs.

	Treatment						*p* Value
	Control	CEM	110-°C ECEM	115-°C ECEM	120-°C ECEM	125-°C ECEM	
T-AOC (U/mL)	5.67 ± 1.17 ab	6.10 ± 0.67 a	5.15 ± 0.73 ab	4.90 ± 0.44 b	6.04 ± 0.44 a	3.18 ± 0.47 c	<0.001
MDA (nmol/mL)	4.38 ± 1.05 c	4.90 ± 1.38 c	8.65 ± 1.57 ab	10.00 ± 1.23 a	9.17 ± 0.76 ab	7.60 ± 0.92 b	<0.001
GSH-Px (U/mL)	478.33 ± 52.44 a	375.56 ± 26.36 c	458.33 ± 25.75 a	398.33 ± 25.23 bc	429.44 ± 27.06 ab	313.89 ± 28.42 d	<0.001
SOD (U/mL)	12.67 ± 2.05	14.62 ± 1.76	12.46 ± 1.09	11.68 ± 1.57	12.02 ± 1.43	13.60 ± 0.83	0.114
CAT (U/mL)	1.44 ± 0.19 b	2.21 ± 0.50 a	2.30 ± 0.21 a	1.74 ± 0.50 ab	1.39 ± 0.23 b	1.58 ± 0.37 b	0.007

Values are expressed as means ± standard deviation (SD) of determinations. One pig was randomly selected from each replicate for blood sampling, with four pigs per treatment in total. a–d Within a row, means without a common superscript letter significantly differ (*p* < 0.05).

**Table 10:** Alpha diversity index of faecal microorganisms in nursery pigs.

	Treatment						*p* Value
	Control	CEM	110-°C ECEM	115-°C ECEM	120-°C ECEM	125-°C ECEM	
ACE	1329.39 ± 41.22	1324.51 ± 171.72	1458.48 ± 76.21	1196.18 ± 167.94	1406.74 ± 62.75	1426.34 ± 61.31	0.118
Chao1	1282.82 ± 37.54	1296.46 ± 175.08	1412.05 ± 76.95	1155.89 ± 154.04	1367.14 ± 68.70	1387.45 ± 70.99	0.120
Simpson	0.97 ± 0.01	0.98 ± 0.01	0.99 ± 0.00	0.98 ± 0.00	0.98 ± 0.00	0.99 ± 0.00	0.110
Shannon	7.00 ± 0.45	7.17 ± 0.56	7.84 ± 0.15	7.33 ± 0.08	7.45 ± 0.18	7.51 ± 0.07	0.057

Values are expressed as means ± standard deviation (SD) of determinations. A total of 528 growing pigs (4 pens per treatment and 22 pigs per pen) with an average initial BW of 13.87 kg. Faecal samples were randomly collected from 3 pigs per treatment for microbial analysis. The same samples were used for subsequent analysis.

**Table 11:** Nursery pigs’ faecal microbial abundance at the phylum level (%).

	Treatment						*p* Value
	Control	CEM	110-°C ECEM	115-°C ECEM	120-°C ECEM	125-°C ECEM	
*Firmicutes*	64.66 ± 4.82	56.51 ± 4.58	62.27 ± 1.99	63.61 ± 5.92	54.91 ± 1.85	59.24 ± 6.01	0.111
*Bacteroidota*	27.36 ± 1.50	33.44 ± 2.52	27.87 ± 1.61	31.97 ± 6.26	35.47 ± 1.74	31.78 ± 4.15	0.086
*Spirochaetota*	4.95 ± 3.24	5.57 ± 3.97	5.25 ± 1.53	1.10 ± 0.46	5.89 ± 2.91	4.27 ± 0.82	0.272
*Desulfobacterota*	1.09 ± 0.65	2.30 ± 1.88	2.03 ± 0.74	1.52 ± 0.70	1.79 ± 0.79	2.19 ± 1.24	0.756

Values are expressed as means ± standard deviation (SD) of determinations. Faecal samples were randomly collected from 3 pigs per treatment for microbial analysis.

**Table 12:** Nursery pigs’ faecal microbial abundance at the genus level.

	Treatment						*p* Value
	Control	CEM	110-°C ECEM	115-°C ECEM	120-°C ECEM	125-°C ECEM	
*Porphyromonadaceae _bacterium*	9.52 ± 2.31	6.92 ± 6.16	3.12 ± 2.36	3.55 ± 0.56	6.82 ± 1.92	5.55 ± 2.53	0.205
*Prevotellaceae*	3.67 ± 1.45	3.49 ± 0.95	5.18 ± 0.91	4.86 ± 1.30	5.62 ± 2.96	6.14 ± 2.99	0.484
*Lachnospiraceae*	4.82 ± 1.33	4.49 ± 2.08	4.12 ± 0.34	5.05 ± 0.69	4.38 ± 1.38	5.24 ± 1.06	0.883
*Lactobacillus*	5.20 ± 4.05	5.24 ± 1.78	3.92 ± 1.01	5.80 ± 2.41	4.33 ± 0.75	2.51 ± 2.09	0.683
*Treponema*	4.93 ± 3.24	5.54 ± 3.96	5.17 ± 1.56	1.08 ± 0.45	5.81 ± 2.95	4.23 ± 0.83	0.281
*Bacilli*	3.77 ± 2.80	3.79 ± 1.53	3.84 ± 1.15	5.11 ± 1.27	3.96 ± 2.01	2.99 ± 1.87	0.836
*Prevotellaceae _NK3B31_group*	2.84 ± 1.20	2.78 ± 0.59	3.90 ± 1.11	4.09 ± 1.37	2.75 ± 0.80	3.10 ± 0.64	0.414
*Streptococcus*	6.79 ± 6.34	2.96 ± 1.81	1.09 ± 0.60	3.27 ± 1.96	0.98 ± 1.02	2.72 ± 1.12	0.297
*Prevotella*	3.49 ± 2.34	5.78 ± 3.63	2.32 ± 0.87	6.22 ± 2.56	6.85 ± 3.62	7.38 ± 2.12	0.220

Values are expressed as means ± standard deviation (SD) of determinations. Faecal samples were randomly collected from 3 pigs per treatment for microbial analysis.

## Data Availability

The data presented in this study are available on request from the corresponding author. In addition, the raw 16S rRNA amplicon sequencing datasets have been deposited in the NCBI Sequence Read Archive (SRA) under BioProject accession PRJNA1509982 and SRA Study accession SRP725470, with BioSample accessions SAMN62282252–SAMN62282269 and SRA Run accessions SRR40084899–SRR40084916.

## Abbreviations

The following abbreviations are used in this manuscript:

AA	Amino acid
ADF	Acid detergent fibre
BCS	Body condition score
BW	Body weight
DM	Dry matter
NDF	Neutral detergent fibre
CEM	*Cyperus esculentus* meal
ECEM	Extruded *Cyperus esculentus* meal
IDF	Insoluble dietary fibre
SDF	Soluble fractions
WHC	Water-holding capacity
OHC	Oil-holding capacity
SC	Swelling capacity
IBW	Initial body weight
FBW	Final body weight
ADG	Average daily gain
ADFI	Average daily feed intake
FCR	Feed conversion ratio
AIA	Acid-insoluble ash
TP	Total protein
GLU	Glucose
ALT	Alanine aminotransferase
ALP	Alkaline phosphatase
BUN	Blood urea nitrogen
